# Rett Syndrome: A Focus on Gut Microbiota

**DOI:** 10.3390/ijms18020344

**Published:** 2017-02-07

**Authors:** Elisa Borghi, Francesca Borgo, Marco Severgnini, Miriam Nella Savini, Maria Cristina Casiraghi, Aglaia Vignoli

**Affiliations:** 1 Department of Health Sciences, Università degli Studi di Milano, 20142 Milan, Italy; francesca.borgo@unimi.it; 2 Institute of Biomedical Technologies, National Research Council, 20090 Segrate, Italy; marco.severgnini@itb.cnr.it; 3 Child Neuropsychiatry Unit, Santi Paolo Carlo Hospital, 20142 Milan, Italy; miriam.savini@unimi.it; 4 Department of Food, Environmental and Nutritional Sciences, Università degli Studi di Milano, 20133 Milan, Italy; maria.casiraghi@unimi.it

**Keywords:** Rett syndrome, microbiota, short-chain fatty acids, diet

## Abstract

Rett syndrome (RTT) is an X-linked neurodevelopmental disorder affecting 1 in 10,000 live female births. Changes in microbiota composition, as observed in other neurological disorders such as autism spectrum disorders, may account for several symptoms typically associated with RTT. We studied the relationship between disease phenotypes and microbiome by analyzing diet, gut microbiota, and short-chain fatty acid (SCFA) production. We enrolled eight RTT patients and 10 age- and sex-matched healthy women, all without dietary restrictions. The microbiota was characterized by 16S rRNA gene sequencing, and SCFAs concentration was determined by gas chromatographic analysis. The RTT microbiota showed a lower α diversity, an enrichment in *Bacteroidaceae*, *Clostridium* spp., and *Sutterella* spp., and a slight depletion in *Ruminococcaceae*. Fecal SCFA concentrations were similar, but RTT samples showed slightly higher concentrations of butyrate and propionate, and significant higher levels in branched-chain fatty acids. Daily caloric intake was similar in the two groups, but macronutrient analysis showed a higher protein content in RTT diets. Microbial function prediction suggested in RTT subjects an increased number of microbial genes encoding for propionate and butyrate, and amino acid metabolism. A full understanding of these critical features could offer new, specific strategies for managing RTT-associated symptoms, such as dietary intervention or pre/probiotic supplementation.

## 1. Introduction

Rett syndrome (RTT; OMIM 312750) is an X-linked neurodevelopmental disorder and one of the most common causes of intellectual disability in females. 90%–95% percent of cases are associated with mutations in the *MECP2* (Methyl CpG Binding Protein 2) gene, encoding a chromatin-associated protein that can both activate and repress transcription [1,2]. RTT is characterized by 6–18 months of apparently normal neurodevelopment followed by neurological regression [3]. Neurological features such as microcephaly, stereotyped hand movements, behavioral problems, seizures, and dyspraxia are the main characteristics of RTT, but respiratory abnormalities and gastrointestinal dysfunctions are also commonly reported [4]. RTT female patients, who are generally heterozygous for *MECP2* gene mutations, have variable MeCP2 expression (and varying phenotypes) due to random X-inactivation patterns. Males who are null for MeCP2 expression are more severely affected and often do not survive birth.

Of the two MeCP2 protein isoforms generated by alternative splicing of the *MECP2* gene (MeCP2E1 and MeCP2E2), MeCP2E1 is the major isoform in the brain in both mice and humans, and its deficiency is responsible for the phenotype [5].

Since MeCP2 is highly expressed in neurons, its loss and/or reduction might affect central nervous system (CNS) development and function [6]. MeCP2 seems to act as a transcriptional modulator rather than a classical transcriptional repressor [7], and *MECP2* mutation type has been proposed as a strong predictor of disease severity [8,9].

Recently, a MeCP2-dependent deregulation mechanism in the enteric nervous system (ENS) has been demonstrated in a *Mecp2*-KO mouse model [10], highlighting ENS plasticity abnormalities similar to CNS. Indeed, girls and women with RTT are characterized by an altered gastrointestinal homeostasis and hypomotility that results in gastrointestinal discomfort, posing a significant burden for their caregivers [11].

Recent results revealed the occurrence of an intestinal sub-inflammatory status in RTT and a concomitant alteration of relative abundances of bacterial and fungal component in RTT subjects compared with healthy controls [12]. Changes in microbiota composition, as observed in other neurological disorders such as autism spectrum disorders (ASD) [13], may contribute to several typical symptoms associated with RTT syndrome.

It has been shown that a dysbiotic gut microbiota may affect the function of the nervous system. Putative mechanisms by which bacterial products affect central and enteric nervous systems are via cytokine release from mucosal immune cells, via the release of gut hormones from enteroendocrine cells, or via afferent neural pathways, including the vagus nerve [14]. This communication is bidirectional: microbiota influences CNS function, and the CNS influences the microbiota composition through its effects on the gastrointestinal tract [15].

Microbial short chain fatty acids (SCFAs), primarily acetate, butyrate, and propionate, are key players in this landscape. Their production relies on various factors such as the microbial species inhabiting the gut, the substrate source and availability, and the gut transit time [16].

Diet can have a marked impact on all the abovementioned factors [17,18], shaping the host microbiota, providing a variety of fermentation substrates, and increasing or reducing fecal bulk and its transit.

To date there has been no clear rationale for dietary intervention and/or pre-/probiotic supplementation to improve gastrointestinal and neurophysiological symptoms in RTT patients.

Recently, RTT diet supplementation with ω-3 polyunsaturated fatty acids was found to improve the patient’s subclinical inflammatory status, partially restoring membrane fatty acids and correct redox status [19].

At the same time, docosahexaenoic acid, a long-chain ω-3 polyunsaturated fatty acid, was shown to promote changes in the gut microbial populations, and, in turn, some microbial genera such as *Bifidobacterium* were demonstrated to improve the tissue distribution of docosahexaenoic acid, especially in the brain [20].

Hence, the diet–microbiota–gut–brain pathway seems to represent a valid target of investigations in order to mitigate or ameliorate disease progression.

In the present work, we characterized gut microbiota and fecal microbial metabolites in RTT patients under unrestricted diet, as well as the correlation between these two players in RTT pathology.

## 2. Results

### 2.1. Cohort Description

We enrolled eight RTT female patients (mean age 23 ± 8.7 years) with different degrees of clinical severity and identified the *MECP2* gene mutation (Table 1).

Their clinical phenotype was classified as classic (C; *n* = 7) or congenital (Co; *n* = 1). The mean score of clinical severity using a modified severity score was 8 (range 5–12). Severity Global Score (SGS) 4–6 was considered as mild phenotype, 7–9 as intermediate, and 10–12 as severe.

All but three subjects were able to walk independently; hand stereotypies were evident in all. Six patients showed useful non-verbal communication, mainly through eye contact.

Epilepsy was diagnosed in seven subjects; three of them still had seizures, while four were seizure-free. Seven patients were taking antiepileptic drugs: two patients were on monotherapy (valproic acid and carbamazepine, respectively), whereas five were on polytherapy (three including valproic acid, two including carbamazepine).

Gastrointestinal discomfort and constipation were present in all subjects, despite regular feeding ability.

As a control group (controls, CTR), we included 10 mentally and physically healthy age-matched women free of any medication (mean age 24.5 ± 6.6 years). The control group was not constipated.

All participants in the study did not take antibiotics or probiotics in the three months before the enrollment, and were Caucasian, living in Northern Italy, and without any diet restriction.

Body mass index (BMI) was calculated using the formula: weight (kg)/height (m)^2^. BMI was 17.2 ± 3.9 (mean ± SD) in RTT patients, and 20.9 ± 2.2 in control group (*p* = 0.073).

### 2.2. Diet Evaluation

Since diet is one of the major environmental factors shaping gut communities, we analyzed the dietary habits of the enrolled subjects. Dietary intake of energy, macronutrients, fiber, and cholesterol is shown in Table 2, in comparison with “reference values” reported in the Nutrients and Energy Reference Intake Levels for the Italian Population (LARN) [21].

We did not observe differences (*p* = 0.291) in the mean value of daily energy intake between RTT patients and controls; for both groups daily energy was lower than the average requirement (AR) reported by (LARN) [21].

Compared with controls, RTT diets evidenced significantly higher level of proteins (*p* = 0.033), exceeding the AR recommended. In particular, RTT diets were characterized by a higher content of animal proteins (*p* = 0.062).

Carbohydrate intake (% of total energy) was significantly lower in RTT (*p* = 0.001), even though about 75% of patients were within the recommended range.

Dietary fiber intake was also decreased in RTT patients (*p* = 0.036), and below the recommended values.

### 2.3. Microbiota Dysbiosis in Rett syndrome (RTT) Patients Is Related to Disease Severity

The gut microbiota was characterized by next-generation sequencing using V3–V4 hyper-variable 16S rRNA genomic region.

On average, 90,390 ± 8648 high-quality reads were considered for gut microbiota analysis of RTT (*n* = 8) and healthy controls (*n* = 10); reads were then grouped in a total of 3742 ± 1552 operational taxonomic units (OTUs), which could be assigned to specific taxonomies down to the genus level.

The gut microbiota composition of the dataset (Figure S1) was dominated by bacteria belonging to *Firmicutes* and *Bacteroidetes* (totaling about 90% of the total average relative abundance); the subdominant phyla were *Proteobacteria* and *Verrucomicrobia* (4.8% and 3.1%, respectively). This profile, even considering the alterations due to RTT, can be considered to be within the limits of a typical Western-style microbiome. At the family level, the dominant groups were *Bacteroidaceae*, *Ruminococcaceae*, and *Lachnospiraceae* (average relative abundance: 30.3%, 13.8%, and 13.0%, respectively), followed by *Rikenellaceae* (8.7%), *Veillonellaceae* (7.8%), and *Porphyromonadaceae* (4.7%).

Sample R4, corresponding to one of our RTT patients, was characterized by a very low biodiversity, as evident from the analysis of cumulative relative abundances at family level (Figure S2A,B), which pointed out that in this sample 97% of the total relative abundance was due to only three families (*Bacteroidaceae*, *Ruminococcaceae*, and *Lachnospiraceae*), whereas other samples (even in the RTT group) reached about 90% at most. This is also reflected in the very low α-diversity values for this sample (Figure S2C,D). Moreover, the collected sample was characterized by liquid consistency (type 7 on the Bristol Stool Scale [22]) and by a SCFA concentration below the detection limits, confirming that it represents a clear outlier in our dataset. All other collected RTT samples were of solid consistency (type 2/3 on Bristol Stool Scale). Thus, we decided to remove it from any further evaluation.

**Figure 1:**
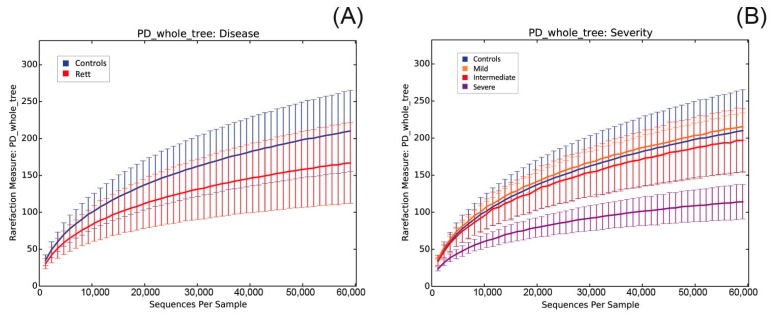
α rarefaction curves according to Faith’s phylogenetic diversity index. (**A**) α-diversity plot of Rett (red) versus control (blue) samples. RTT patients show a reduced biodiversity compared to healthy controls. Differences are not statistically significant (*p* > 0.05); (**B**) Data grouped according to disease severity. Severe stage (purple) samples show a reduced biodiversity compared to healthy controls (blue), mild (orange), or intermediate (red) disease stage. Differences are not statistically significant (*p* > 0.05).

Bacterial composition within each sample (α-diversity) was measured using OTU-based methods (Chao1, Figure S3A,B; observed species, Figure S3C,D; and Shannon indexes, Figure S3E,F), and phylogenetic tree-based (PD-WT) method (Figure 1). First, we compared differences between RTT patients and controls subjects (Figure 1A). Secondly, we examined the effect of disease severity on bacterial communities (Figure 1B).

Even though we could observe a reduction in α-diversity in RTT patients, the number of subjects analyzed was not sufficient to reach statistical significance (*p* > 0.05, permutation-based non-parametric *t*-test) for all metrics. The observed reduction was more pronounced in patients with a severe form of the disease.

**Figure 2:**
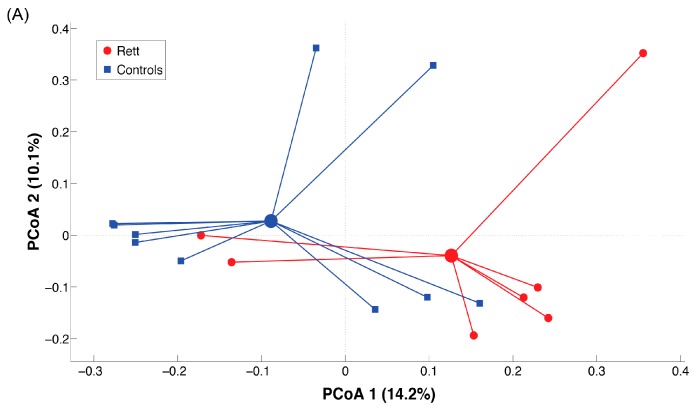

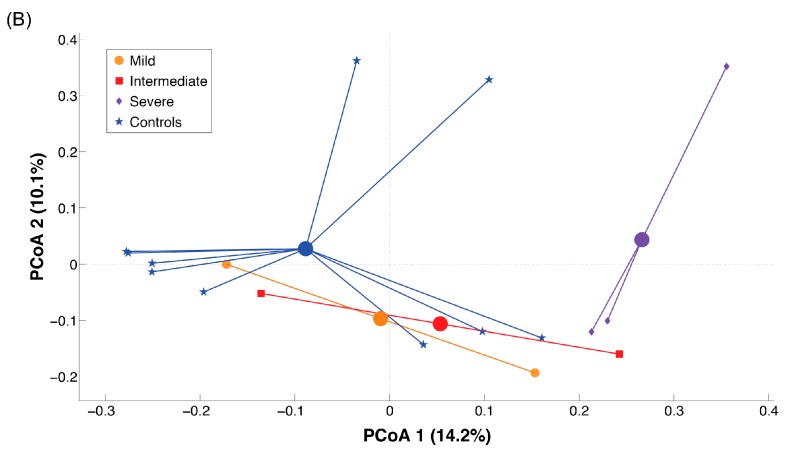
Principal Coordinate Analysis (PCoA) according to unweighted Unifrac distance. The first two components of the variance are represented. (**A**) PCoA of Rett (red) versus control (blue) samples. The two groups tend to separate according to PCoA 1 component. Differences are not statistically significant (*p* > 0.05); (**B**) Samples are colored according to disease severity. Severe stage (purple) significantly (*p* < 0.05) separates from healthy controls. Other differences are not statistically significant (*p* > 0.05).

To investigate differences in the two studied groups, we assessed β-diversity using unweighted (Figure 2) and weighted UniFrac distances (Figure S4).

β-diversity analysis highlighted a certain separation (although not statistically significant) between the centroids of RTT and CTR groups according to the unweighted Unifrac distance (*p* = 0.06). Moreover, classification according to disease severity highlighted how severe forms of RTT result in a completely different microbiota signature, as evidenced by the separation between severe stage RTT patients and CTR (*p* = 0.02). These differences are likely due to subdominant components of the gut microbiota, as also suggested by the fact that separation on weighted Unifrac metric is not significant (Figure S4).

To evaluate possible differences in taxa distribution amongst RTT and control subjects, we analyzed the relative microbial abundance at different taxonomic levels.

**Figure 3:**
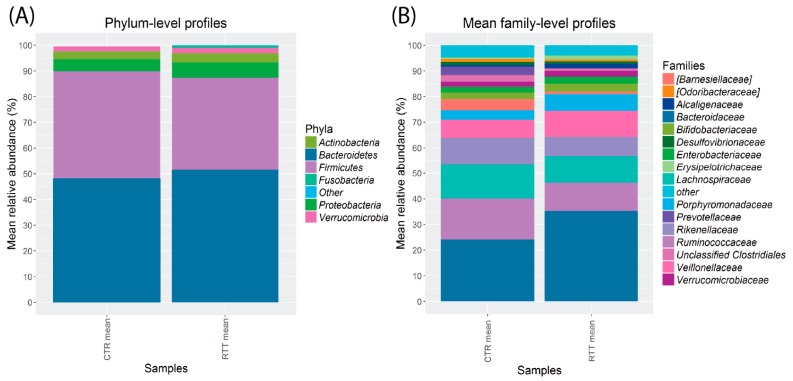
Bar charts representing the average relative abundance of Rett (RTT, *n* = 8) and control (CTR, *n* = 10) microbiota, classified using the 16S rRNA gene. (**A**) Mean relative abundances of fecal bacterial phyla; (**B**) Mean relative abundances of fecal bacterial families.

At the phylum level (Figure 3A), the predominant bacterial taxa in feces of both RTT and CTR subjects were *Bacteroidetes* (CTR: 48.1 ± 19.2, RTT: 51.5 ± 11.2; mean ± sd) and *Firmicutes* (CTR: 41.9 ± 18.7, RTT: 35.9 ± 10.7), followed by *Proteobacteria* (CTR: 4.6 ± 4.3, RTT: 5.8 ± 5.0), *Actinobacteria* (CTR: 3.0 ± 4.2, RTT: 3.6 ± 3.9), and *Verrucomicrobia* (CTR: 2.0 ± 2.6, RTT: 2.3 ± 3.4). The gut microbiota of RTT patients was characterized by a slight increase in *Bacteroidetes* (+3.4% on average) and a decrease in *Firmicutes* (−6.0% on average).

The most abundant families (Figure 3B) were *Bacteroidaceae* (CTR: 23.9 ± 5.6, RTT: 35.3 ± 7.4; mean ± sd) *Ruminococcaceae* (CTR: 17.8 ± 10, RTT: 11.5 ± 11.7), *Lachnospiraceae* (CTR: 14.9 ± 9.6, RTT: 10.8 ± 10.6), *Rikenellaceae* (CTR: 9.5 ± 13.8, RTT: 8.6 ± 9.6), and *Veillonellaceae* (CTR: 7.2 ± 6.2, RTT: 10.9 ± 6.2).

RTT microbiota were enriched in *Bacteroidaceae* (as well as *Bacteroides*, *p* < 0.05), *Clostridium* spp. (*p* < 0.01), *Sutterella* spp., and slightly depleted in *Ruminococcaceae* (as well as *Faecalibacterium prasunitzii*), *Prevotella* spp., and *Roseburia* spp.

We observed for some taxa a severity-related relative abundance: *Bacteroidaceae*, *Enterobacteriaceae*, and *Erysipelotrichaceae* increased, whereas *Ruminococcaceae* decreased from mild to severe disease (Figure S5).

In order to determine whether body mass index could impact on intestinal microbiota ecology, as observed in a previous study [23], we evaluated the relationship between BMI and microbiota composition. BMI was positively correlated with *Ruminococcaceae* (*p* = 0.044), and inversely correlated with *Bacteroidaceae* (as well as *Bacteroides*, *p* = 0.0174) and *Veillonaceae* abundance (*p* = 0.0472).

Correlation analysis between diet and microbiota showed that *Bacteroides* and *Clostridium*, significantly increased in RTT, positively correlated with total protein and animal protein intake (*p* < 0.05), whereas fiber intake was positively correlated with *Christensenellaceae*, and slightly increased in the CTR group (Table S1).

### 2.4. Microbial Metabolites Are Influenced by Diet and by a Shift in Some Microbial Populations

Changes in microbial species could alter the amounts of microbial metabolites, in particular short-chain fatty acids (SCFAs), produced as fermentation products from food components that are unabsorbed/undigested in the small intestine.

Acetate, butyrate, and propionate are mainly derived from carbohydrate fermentation, whereas branched-chain fatty acids (BCFAs, 5% of total SCFAs), mainly *iso*-butyrate and *iso*-valerate, are from proteins and amino acid fermentation by proteolytic bacteria [16].

**Figure 4:**
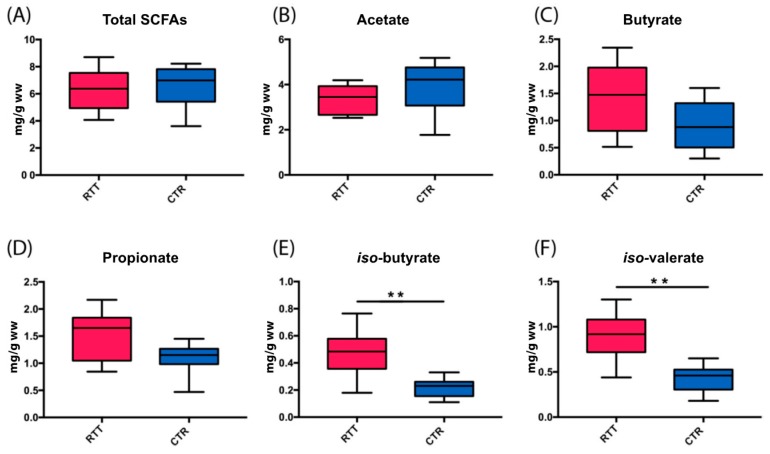
Box-and-whisker plots of fecal microbial metabolites in feces of Rett (RTT, magenta) and control (CTR, blue) subjects: (**A**–**D**) short-chain fatty acids (SCFAs); (**E**,**F**) branched-chain fatty acids (BCFAs). Data refer to mg/g wet weight (mg/g ww) of feces. Significant differences are indicated by ** (*p* < 0.01), Mann–Whitney test.

Total SCFAs fecal content (Figure 4A) was similar in the two experimental groups (*p* = 0.387, Mann–Whitney test) as well as acetate (*p* = 0.112, Figure 4B). Butyrate and propionate concentrations (Figure 4C,D) were increased in RTT patients (*p* = 0.073). SCFAs percentages ratio (Acetate:Propionate:Butyrate) was 72:17:12 in controls and 61:22:17 in RTT subjects. Fecal BCFAs (Figure 4E,F) were significantly higher in the RTT population (*p* < 0.008 and *p* < 0.006 for *iso*-butyrate and *iso*-valerate, respectively).

Total SCFAs and acetate concentrations were positively correlated with the subject’s body mass index (*p* = 0.029 and *p* = 0.021, respectively). A similar trend was observed for acetate (*p* = 0.021), which was slightly increased in the CTR group compared with the RTT group. No correlation was seen for butyrate and propionate.

**Figure 5:**
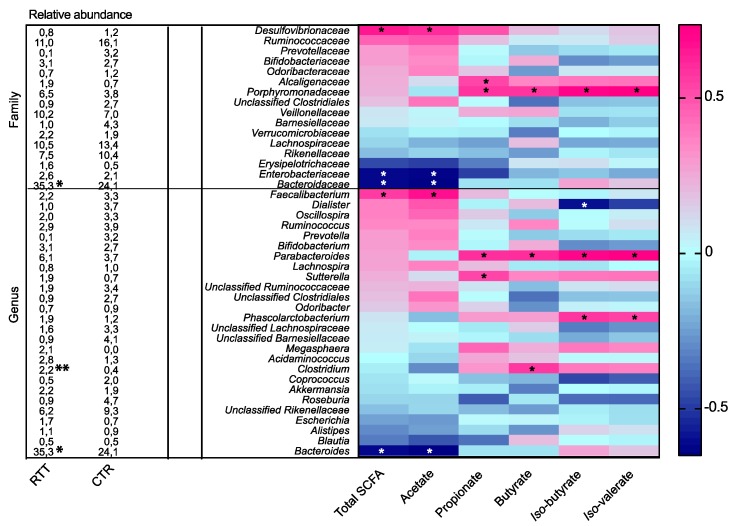
Correlations between intestinal family- and genus-level phylogenetic groups and fecal short-chain fatty acids (SCFAs) (heatmap). **Right** panel: correlations between intestinal bacteria and SCFAs are indicated by colors (magenta: positive; blue: negative). Asterisks (*) indicate statistically significant correlations (*p* < 0.05); **Left** panel: mean relative abundances in RTT and CTR are reported. Significant differences are indicated by * *p* < 0.05, and ** *p* < 0.01, Mann–Whitney test.

We then evaluated possible associations between SCFA concentration and bacterial populations (Figure 5).

The *Bacteroidaceae* family showed an inverse and significant correlation (*p* < 0.05) with total SCFAs and acetate concentration, and seemed to slightly contribute to BCFA production. A similar trend was observed at the genus level for *Bacteroides* spp., whereas *Parabacteroides*, a saccharolytic genus belonging to *Bacteroidetes*, was positively correlated with propionate, butyrate, and BCFA concentrations (*p* < 0.05). *Alcaligenaceae* is positively correlated with propionate, whereas *Porphyromonadaceae* is positively correlated with propionate, butyrate, and BCFA concentration (all *p* < 0.05).

All the abovementioned taxa were increased in RTT subjects and could be related to the different SCFAs concentrations observed.

Moreover, RTT gut communities showed a reduction of *Ruminococcaceae*, and in particular of *Faecalibacterium* spp., the latter significantly correlating with total SCFAs and acetate production (*p* < 0.05).

**Figure 6:**
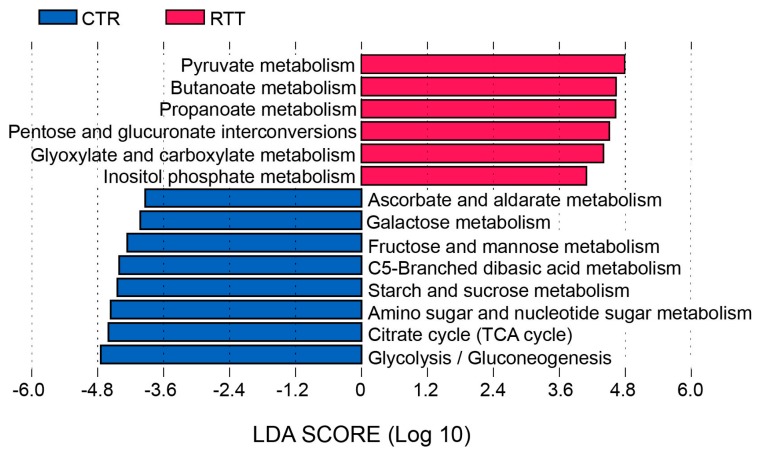
Functional characterization of carbohydrate metabolism, differentially represented in the gut microbiota of RTT and control subjects. Kyoto Encyclopedia of Genes and Genomes (KEGG) pathways have been identified by linear discriminant analysis coupled with effect size (LEfSe) (LDA  >  2, *p* < 0.05). Magenta histograms: pathways enriched in RTT patients; blue histograms: pathways enriched in control subjects.

Metabolic pathways are highly redundant in the gut microbiome [24], with several genera participating in carbohydrate and/or protein fermentation, and metabolite production. We applied PICRUSt (Phylogenetic Investigation of Communities by Reconstruction of Unobserved States) analysis to predict possible pathways enriched or depleted in RTT bacterial communities (Figure S6). At a broad functional level, the analysis predicted an enrichment in genes encoding enzymes for carbohydrate and lipid metabolism in the microbiota of healthy subjects, whereas the amino acids pathway was increased in RTT patients. However, genes for butanoate and propanoate metabolism were increased in the RTT microbial community (Figure 6).

## 3. Discussion

Our study showed changes in the intestinal microbial profile that could affect comorbidities associated with RTT syndrome. This is the second study investigating the RTT microbiome and the first, to our knowledge, that compared dietary intakes, anthropometric measurements, microbial profiles, and fecal SCFA concentrations.

Our data suggest that the RTT microbial population is reduced in richness and evenness. Decreasing in α diversity directly correlated with disease phenotype: patients with higher scores of clinical severity showed lower microbial diversity. Despite the small cohort enrolled, our results are in agreement with what was observed by Strati and colleagues for the same syndrome [12], and with other authors on subjects affected by ASD [25]. A rich microbial ecology promotes the fundamental crosstalk and cross-feeding between species that guarantees the stability and resilience of the gut ecosystem; loss of diversity is a consistent trait of intestinal dysbiosis [26].

In our RTT cohort we found a slight reduction in *Firmicutes* and an increase in *Bacteroidetes*, showing a pro-inflammatory status of the gut microbiota, in accordance with what was already reported in studies on inflammatory bowel disease [27]. Interestingly, other neurological disorders, such as Parkinson’s disease, also seem to be characterized by a similar gut microbiota profile [28]. Strati and colleagues observed an inverse trend in their RTT cohort. As previously described in both children and adults [23,29], we found an inverse correlation between the relative abundance of *Bacteroidetes* and body mass index (BMI). It is thus possible that the two studied cohorts were affected by BMI variations justifying different trends.

On the other hand, our study confirmed an increase in RTT patients of *Erysipelotrichaceae*, and at the genus level of *Clostridium* spp., *Sutterella* spp., and *Escherichia* spp. [12].

*Erysipelotrichaceae* have been suggested to be linked to gut inflammation, and to lipidemic imbalance [30], features that have been reported in RTT syndrome [12,19], and our data indicate a severity-related increase in abundance.

*Bifidobacterium* spp.’s relative abundance was similar in RTT and CTR subjects, irrespective to the disease status, without confirming its overgrowth in RTT patients [12]. Our cohort was older than the patients enrolled by Strati et al. (mean age 23 years compared with 12), and bifidobacteria abundance is inversely correlated with age, and strongly dependent on diet [31,32,33]. An increase in bifidobacteria has not been associated with any disease status, whereas a decrease has been observed in allergic children, obese subjects, and the elderly [34].

Diet is the major force shaping the gastrointestinal microbiota, and differences in macronutrient intake can promote the overgrowth of selected microbial taxa [33]. Diet provides a variety of substrates for bacterial fermentation, and consequently can alter microbial metabolite types and concentration. Despite feeding difficulties, patients with RTT do not have any dietary restrictions [11]. In the RTT group, we recorded higher protein consumption, mainly due to higher animal protein intake, and a lower fiber intake in comparison with healthy controls, thus indicating different dietary patterns between the two groups. Dietary intakes analysis showed that proteins, carbohydrates (% of total energy), and dietary fiber intake were lower than those reported by LARN [21] in all the RTT subjects. In the present study, reference values for healthy people were used as, to the best of our knowledge, no dietary recommendations for RTT patients have been reported.

Further studies are needed to assess nutritional requirements in order to establish a dietetic approach for these patients.

The above-described differences in microbiota communities did not result in differences in the total fecal concentration of SCFAs, which was similar in all participants in our study, suggesting that total colonic fermentation does not differ in the two groups. SCFA analysis was performed on wet stool, thus not accounting for their water content. Although with this method SCFAs and BCFAs might be highly concentrated, the literature supports this methodological approach, especially for RTT patients, in view of their known constipation status [12].

In agreement with other studies, fecal total SCFA concentration was positively correlated with BMI [23,29].

We found fecal butyrate and propionate to be increased in RTT patients, as well as *iso*-butyrate and *iso*-valerate.

A functional group of bacteria, including *Lachnospiraceae* and *Ruminococcaceae*, has been identified as responsible for butyrate intestinal concentration [35], but RTT microbiota was depleted in these families. On the other hand, functional analysis with PICRUSt predicted in RTT patients an increase in genes encoding enzymes involved in butanoate and propanoate metabolism. Recently, additional enzymes involved in butyrate synthesis, alternatives to the acetyl-coenzyme A pathway, have been recognized in many other bacterial taxa [36] and could account for the observed butyrate increase. In particular, the lysine pathway indicates that protein, increased in the RTT diet, could also serve in substrate butyrate synthesis [36].

Higher fecal butyrate concentrations could also result from its decreased absorption. Butyrate adsorption, and thus inversely its fecal content, is dependent on gut transit time. A transit >50 h promotes total butyrate oxidation by colonocytes that utilize it as a preferred energy source [37].

Constipation is common in RTT patients, prolonging bulk transit time; it is thus possible that the increase in butyrate fecal content is linked to a reduction due to an altered colonocyte proliferation [38]. Butyrate plays a crucial role in gastrointestinal homeostasis by reducing inflammation, promoting intestinal motility, and stimulating mucin production [39].

RTT subjects also show a slight increase in propionate fecal content, which is in line with a greater abundance of *Bacteroidetes* and *Veillonaceae*, major propionate producers [40]. As observed by other authors, propionate proportion showed a significant positive correlation with the *Bacteroidetes* [41]. Propionate is a substrate for hepatic gluconeogenesis and has been demonstrated to inhibit cholesterol synthesis [16]. Besides the beneficial effects of propionate, its intraventricular direct infusion into rat brains showed neurotoxicity potential, promoting the increase of oxidative stress and neuroinflammation [42].

Protein fermentation is less studied than carbohydrates; however, it has been suggested that branched-chain fatty acids (BCFAs) and other products coming from protein fermentation, such as ammonia, phenols, amines, and sulfides, could affect the viability of colonocytes [43]. PICRUSt analysis predicted higher amino acid metabolism in the RTT group, which is in line with the higher protein intake. BCFAs derive from the degradation of protein, in particular from animal protein rich in branched-chain amino acids, by proteolytic bacteria such as *Bacteroidetes*.

We found RTT microbiota to share many alterations observed in ASD patients, such as enrichment in *Bacteroidaceae*, *Clostridium*, and *Sutterella* [44,45], and reduction in *Prevotella*, and *Coprococcus* [25]. However, RTT patients showed a characteristic microbial signature that lacks the increase in *Desulfovibrionaceae* and reduction in *Veillonellaceae* observed in ASD.

Until the last revision of the Diagnostic and Statistical Manual of Mental Disorders (DSM-V) published in 2013, RTT was included in Pervasive Developmental Disorders [46], because of the absence of spoken language and the stereotyped behaviors. Autistic-like characteristics are mostly evident in the regression phase, whereas in the later phases good eye contact distinguishes RTT patients from ASD subjects. RTT syndrome is now considered a unique disease, with a well-defined genetic background and clinical criteria [47].

Constipation is another common feature of RTT and ASD patients. However, the *MECP2* defect of RTT may itself account for gastrointestinal hypomotility [10]; we cannot rule out that this by itself is responsible for shaping the diverse microbial colonization. It is also possible that genetic defects can be ameliorated by SCFAs-derived compensatory effects. Acetate, and to a lesser extent butyrate and propionate, can increase the colonic blood flow with effects on muscle contraction, tissue oxygenation, and nutrient supply [37].

Drugs can also potentially alter the microbiota. The enrolled RTT patients were on different antiepileptic therapies, but gut characteristics appear to be homogeneous between subjects. On the other hand, we cannot rule out the specific influence of antiepileptic drugs (AEDs) on our results because no data are available about AEDs’ impact on the gut microbiota. Vice versa, intestinal microbiota can alter drug absorption [48], and there is evidence linking some commensal species to the regulation of autoimmune responses triggering CNS inflammation [49].

Further studies are needed and encouraged in this field to identify possible relationships between microbiota and AEDs in patients with epilepsy.

To date, effective therapies to cure RTT by restoring *MECP2* gene function are lacking. At the same time, caregivers are challenged by RTT-derived comorbidities such as constipation, epileptic seizures, and growth retardation.

Unraveling the fine relationship between RTT dysbiosis and specific disease phenotype could offer the chance to study alternative or combined therapies, patient-designed, to improve RTT-associated symptoms and, ultimately, psycho-physical wellness.

Preliminary trials involving children with ASD showed that probiotic supplementation improves antisocial behavior, anxiety, and communication problems. The resolution of gastrointestinal discomfort itself resulted in an improvement of behavioral disturbances [50].

Beside probiotic supplementation, diet intervention, which is easily achievable, could offer a further way to restore or improve microbial species that have been found to have decreased or increased in RTT subjects.

Ketogenic diet interventions, assessed in mouse models of RTT and ASD, resulted in an improvement in motor and social behavior [51]. Beneficial effects have also been obtained with choline-rich [52] or anaplerotic triheptanoin diets [53].

A comprehensive study of a bigger cohort of RTT individuals, combining nutritional intervention with detailed information on its effects on microbial community, microbial metabolites, biochemical parameters, and neurophysiologic patterns could be fundamental to determining the most effective treatment to ameliorate RTT patients’ quality of life.

## 4. Materials and Methods

### 4.1. Subject Recruitment

RTT subjects were enlisted from a cohort of patients referred to the Child Neuropsychiatry Department of Santi Paolo Carlo Hospital (Milan, Italy). The diagnosis of RTT was made according to the new Rett diagnostic criteria defined in 2010 [47]. Phenotypes of patients were categorized as follows: classic (C), congenital variant (Co), and preserved speech variant (PSV) [47]. Inclusion criteria were clinical diagnosis of RTT and demonstrated *MECP2* mutation, while exclusion criteria were the use of antibiotics or probiotics in the three months before. As a control group, we included 10 mentally and physically healthy girls who were not on any medication.

Evaluation of clinical severity included a detailed history and physical examination, and a quantitative measures of global clinical status: a modified Severity Global Score (SGS) [54]. All scores range from 1 to 3, with 1 representing the least severe and 3 representing the most severe cases.

From all subjects we collected stool samples, anthropometric data, and dietary habits. Enrolled patients’ caregivers and control subjects filled in a food diary to calculate daily food intake in kcal/day. Food diaries were processed by dieticians to calculate the average amounts of energy and nutrient intake using commercially available software (MetaDieta^®^, Software version 3.1, METEDA S.r.l., San Benedetto del Tronto, Italy). Anthropometric evaluation included measurements of height and weight; body mass index was calculated and expressed as kg/m^2^.

The study protocol was approved by the Local Ethics Committee (protocol number 2016/ST/199, 28 July 2016); written informed consent was obtained from healthy subjects and from the parents or legal guardians of the enrolled patients.

### 4.2. DNA Extraction and Preparation of 16s rRNA Gene Amplicon Libraries

Total bacterial DNA extraction was performed using the Spin stool DNA kit (Stratec Molecular, Berlin, Germany), according to the manufacturer’s instructions and amplified by PCR. 25 ng of DNA extracted from each stool sample was utilized to construct a sequencing library. 16S rRNA gene amplicon libraries were performed with a two-step barcoding approach according to Illumina 16S Metagenomic Sequencing Library Preparation (Illumina, San Diego, CA, USA). In the first-step PCR, 16S rRNA gene of all bacteria was amplified as described by Klindworth et al. [55]. Then, for library preparation, DNA samples resulting from first PCR step were amplified with dual-index primers using Nextera DNA Library Preparation Kit (Illumina). Each sample possessed specific barcode sequences at the 5′- and 3′-end of the PCR amplicon to discriminate among each other in the pooled library. Library concentration and exact product size were measured using a KAPA Library Quantification Kit (Kapa Biosystems, Woburn, MA, USA) and an Agilent 2100 Bioanalyzer System (Agilent, Santa Clara, CA, USA), respectively.

### 4.3. Sequencing via an Illumina MiSeq Platform

A pooled library (20 nM) and a PhiX control v3 (20 nM) (Illumina) were mixed with 0.2 N fresh NaOH and hybridization buffer HT1 (Illumina) to produce the final concentration at 12 pM each. The resulting library was mixed with the PhiX control v3 (5%, *v*/*v*) (Illumina) and 600 µL loaded on a MiSeq^®^ v2 (500 cycle) Reagent cartridge for obtaining a paired-end 2 × 250 bp sequencing. All sequencing procedures were monitored through the Illumina BaseSpace^®^ application. Demultiplexed FASTQ files were generated by Illumina MiSeq Reporter and a total of 2.5 Gbases were obtained (average of 286,167 reads per sample). Sequencing reads are available in NCBI Short Read Archive (SRA, http://www.ncbi.nlm.nih.gov/sra) under ID PRJNA355083.

### 4.4. Short Chain Fatty Acid (SCFA) Measurement

SCFA concentrations were assessed in accordance with the method proposed by Weaver et al. [56], modified as follows. Stool (200 mg) were suspended in 1 mL of double distilled water, homogenized on a vortex mixer, and, after 30 min, centrifuged (15,000 rpm) for 15 min at 10 °C. Aliquots (0.5 mL) of supernatant were added with 100 µL 85% orthophosphoric acid, 100 µL 2% (*v*/*v*), sulfuric acid and 100 µL of 2-ethyl-butyric acid (109959 Sigma-Aldrich, Milan, Italy) 10 mM in HCOOH 12% as internal standard. SCFA were gently extracted for 1 min with 1 mL ethyl-ether/heptan (1:1 *v*/*v*) and centrifuged for 10 min at 3000 rpm. The aqueous phase was frozen and the organic layer was removed for analysis by a Varian 3400 CX (Conquer Scientific, San Diego, CA, USA) gas liquid chromatograph equipped with a Varian 8200 CX autosampler and an HP-FFAP fused-silica capillary column (30 m, 0.53 mm i.d. with a 1-mm film). Injector and detector temperatures were 110 and 260 °C, respectively. The initial oven temperature was 60 °C and was increased by 10 °C/min to 110 °C and then by 5° C/min and held at 200 °C for 5 min. Quantification of the SCFA was obtained through calibration curves of acetic, propionic, *iso*-butyric, butyric, and *iso*-valeric acid in concentrations between 2.5 and 10 mM (10 mM 2-ethyl-butyric acid as internal standard). Results are expressed as mg/g of wet weight of feces.

### 4.5. Data Analysis

Raw sequencing reads were processed merging together read pairs by using PandaSeq software (Software version 2.5, “PAired-eND Assembler for DNA sequences”) [57], which performs a local assembly between two overlapping pairs, generating a single fragment covering the whole V3–V4 amplicon. Fragments of length <250 bases or >900 bases, as well as non-overlapping sequences, were discarded. Then, fragments were filtered using the “split_libraries_fastq.py” utility of the QIIME suite [58], which filters out sequences having more than 25% nucleotides with a phred score of 3 or less. Quality-filtered reads were then analyzed with the standard QIIME pipeline. Sequences were grouped into OTUs (Operational Taxonomic Units) by using UCLUST [59] with 97% similarity threshold and taxonomically classified against the 13.8 release of the Greengenes bacterial 16S rRNA database (http://greengenes.lbl.gov) by RDP classifier [60] at 50% confidence. Singleton OTUs (i.e., clusters made up of only 1 read) were discarded as possible artifacts or unlikely bona fide bacterial sequences. Sample biodiversity (i.e., α diversity evaluation) was estimated according to different microbial diversity metrics (i.e., chao1, Shannon index, observed species and Faith’s phylogenetic distance).

Inter-sample diversity (i.e., beta-diversity) was calculated using both weighted and unweighted Unifrac metrics [61] and Principal Coordinates Analyses (PCoAs) were conducted. Data separation was tested with a permutation test with pseudo *F*-ratios. For relative abundance analysis, a Mann–Whitney *U*-test was used; a *p*-value <0.05 was chosen as the threshold for statistical significance. All statistical evaluations were performed in Matlab (Software version 7.7.0, Natick, MA, USA).

Phylogenetic Investigation of Communities by Reconstruction of Unobserved States (PICRUSt) 1.0.0 [62] was applied to predict metagenome function from the 16S rRNA data.

We associated OTUs with known bacterial genomes precalculated in PICRUSt by picking closed OTUs against the Greengenes 16S rRNA gene database (13.5). The resulting OTU table was then normalized and used for metagenome inference of Kyoto Encyclopedia of Genes and Genomes (KEGG) Orthologs using PICRUSt. Bray–Curtis distances were used to determine the similarity of samples on the basis of metagenomic composition. Differences in the predicted molecular functions of the bacterial communities of RTT and CTR groups were analyzed by the linear discriminant analysis (LDA) effect size (LEfSe) [63] with default settings on the website https://huttenhower.sph.harvard.edu/galaxy/root.

## 5. Conclusions

Gut microbiota produces an array of bioactive metabolic products that could exert profound and dynamic effects on host metabolism and gene expression in many systems, including the Central and the Enteric Nervous systems. A dysbiotic intestinal microbiota might contribute to or worsen several RTT traits and might represent a possible disease marker and therapy target. Strategies aimed at restoring normal gut microbiota, such as supplementation with pre/probiotics may represent a non-pharmacological option not only for gastrointestinal disturbances, but also for behavioral and neurophysiological abnormalities associated with RTT.

## Figures and Tables

**Table 1:** Genetic defects and disease phenotypes of Rett syndrome (RTT) patients.

Patient	*MECP2* Mutation	Type of Mutation	Disease Phenotype	SGS
R1	p. (R255 *)	Nonsense	Classic	12
R2	p. (L383Pfs)	Frame-shift	Classic	7
R3	c. (27 + 1_28 − 1)_(1461_?)del	Intragenic deletion	Classic	5
R4	p. (R133C)	Missense	Classic	5
R5	p. (R270Efs)	Frame-shift	Classic	10
R6	p. (R306C)	Missense	Classic	6
R7	p. (R294 *)	Nonsense	Classic	8
R8	p. (R106W)	Missense	Congenital	12

SGS: Severity Global Score. * Stop mutation is currently indicated by asterisk.

**Table 2:** Daily macronutrient intake in RTT and control (CTR) individuals.

Variable	RTT Mean ± SD	Control Mean ± SD	*p*-Value	Reference Values (LARN)
Energy				
kcals	1739 ± 680	1493 ± 202	0.291	Women: 1790–2320 kcal (AR)
Proteins				
g	70.3 ± 21.8	51.5 ± 12.2	0.033 *	43–50 g (AR)
g/kg weight	2.0 ± 0.6	1.0 ± 0.3	<0.001 ***	0.71 g/kg bw (AR)
% E	16 ± 4	14 ± 3	0.087	12%–15% E (RI)
Animal proteins				
g	46.3 ± 19.8	31.3 ± 11.9	0.062	
g/kg weight	1.3 ± 0.5	0.6 ± 0.3	<0.001 ***	
Vegetal proteins				
g	17.9 ± 6	20.2 ± 3.3	0.151	
g/kg weight	0.5 ± 0.2	0.4 ± 0.1	0.180	
Total Carbohydrates				
g	181.5 ± 65.9	209.0 ± 25.9	0.242	
g/1000 kcal	107.0 ± 22.0	140.7 ± 13.9	0.001 **	
% E	43 ± 9	56 ± 6	0.001 **	45%–60% E (RI)
Sugars				
% E	13 ± 3	15 ± 4	0.302	<15% E (SDT)
Fiber				
g	11.3 ± 5.4	14.5 ± 5.3	0.225	
g/1000 kcal	6.5 ± 1.6	9.9 ± 3.9	0.036 *	8.4–12.6 g/1000 Kcal
Fats				
g	65.5 ± 15.0	52.9 ± 12.8	0.243	
g/1000 kcal	37.3 ±7.8	35.2 ± 5.2	0.488	
% E	33 ± 7	32 ± 5	0.488	20%–35% E (RI)
Saturated Fats				
% E	8 ± 3	11 ± 4	0.117	<10% E (SDT)
Cholesterol				
mg	244 ± 97	156 ± 66	0.035 *	<300 mg·day

AR, average requirement; RI, reference intake; SDT, suggested dietary target; % E, percentage of total energy. Significant differences are indicated by * *p* < 0.05, ** *p* < 0.01, and *** *p* < 0.001, Mann–Whitney test.

## Supplementary Materials

Supplementary materials can be found at www.mdpi.com/1422-0067/18/2/344/s1.

## Abbreviations

AED	Antiepileptic drug
ASD	Autism spectrum disorders
BCFA	Branched-chain fatty acids
BMI	Body mass index
CNS	Central nervous system
CTR	Controls
ENS	Enteric nervous system
LDA	Linear discriminant analysis
MeCP2	Methyl CpG binding protein 2
OTU	Operational taxonomic units
RTT	Rett syndrome
SCFA	Short-chain fatty acids
SGS	Severity Global Score
