# Supplementary material for: Rett Syndrome: A Focus on Gut Microbiota
Source: Int J Mol Sci. 2017 Feb 7;18(2):344. doi: 10.3390/ijms18020344 (PMC5343879; doi:10.3390/ijms18020344)
Supplement: Supplementary file 1 [file ijms-18-00344-s001.pdf]

Supplementary Materials: Rett Syndrome: A Focus on Gut Microbiota

Elisa Borghi, Francesca Borgo, Marco Severgnini, Miriam Nella Savini, Maria Cristina Casiraghi and Aglaia Vignoli

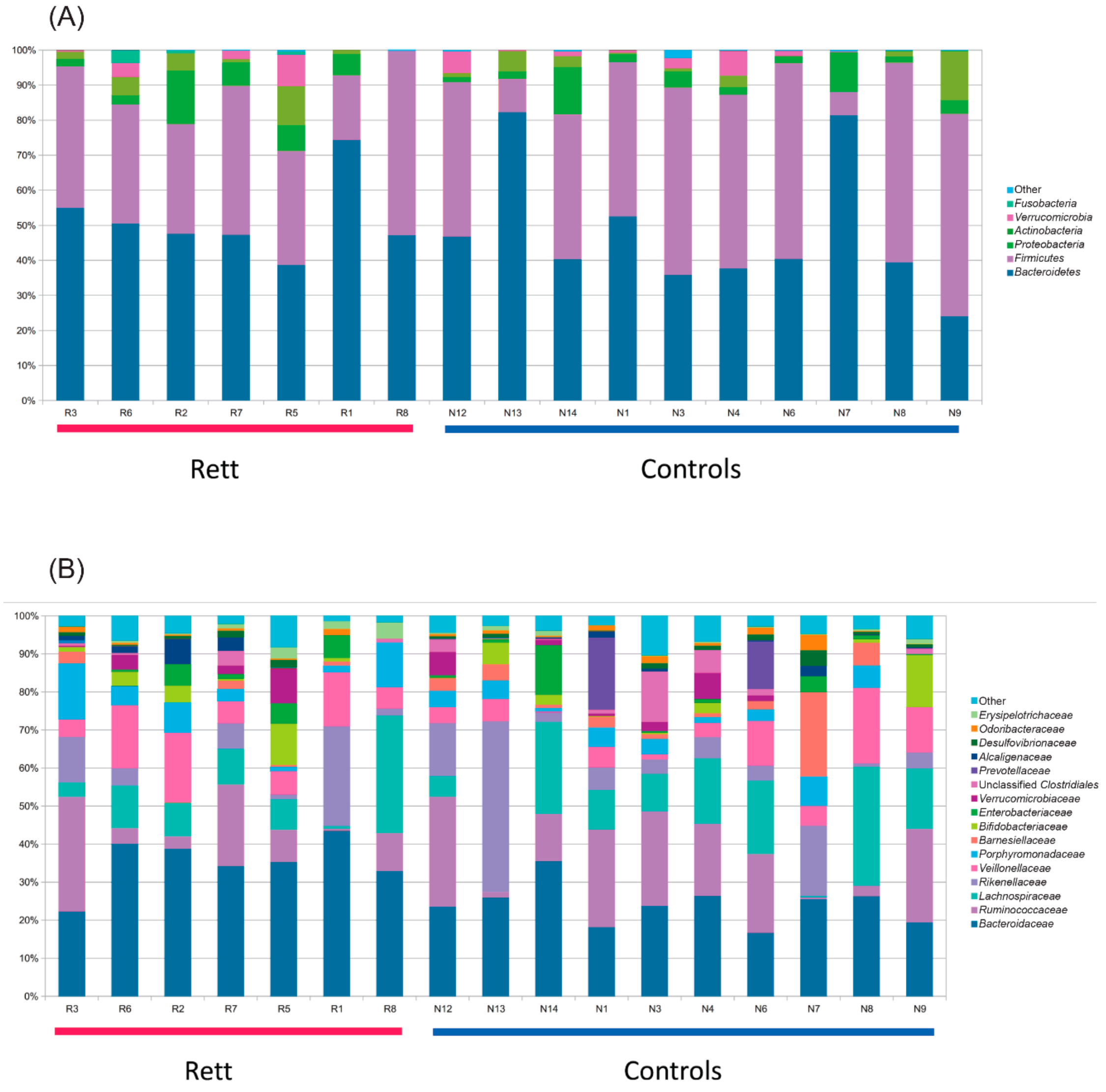

Figure S1. Histograms of relative abundances at phylum (**A**); and family (**B**) level for all the 8 Rett (RTT) and 10 control (CTR) samples. Only groups with a mean relative abundance >1% are represented. Less abundant groups are summed up in the "Other" category.

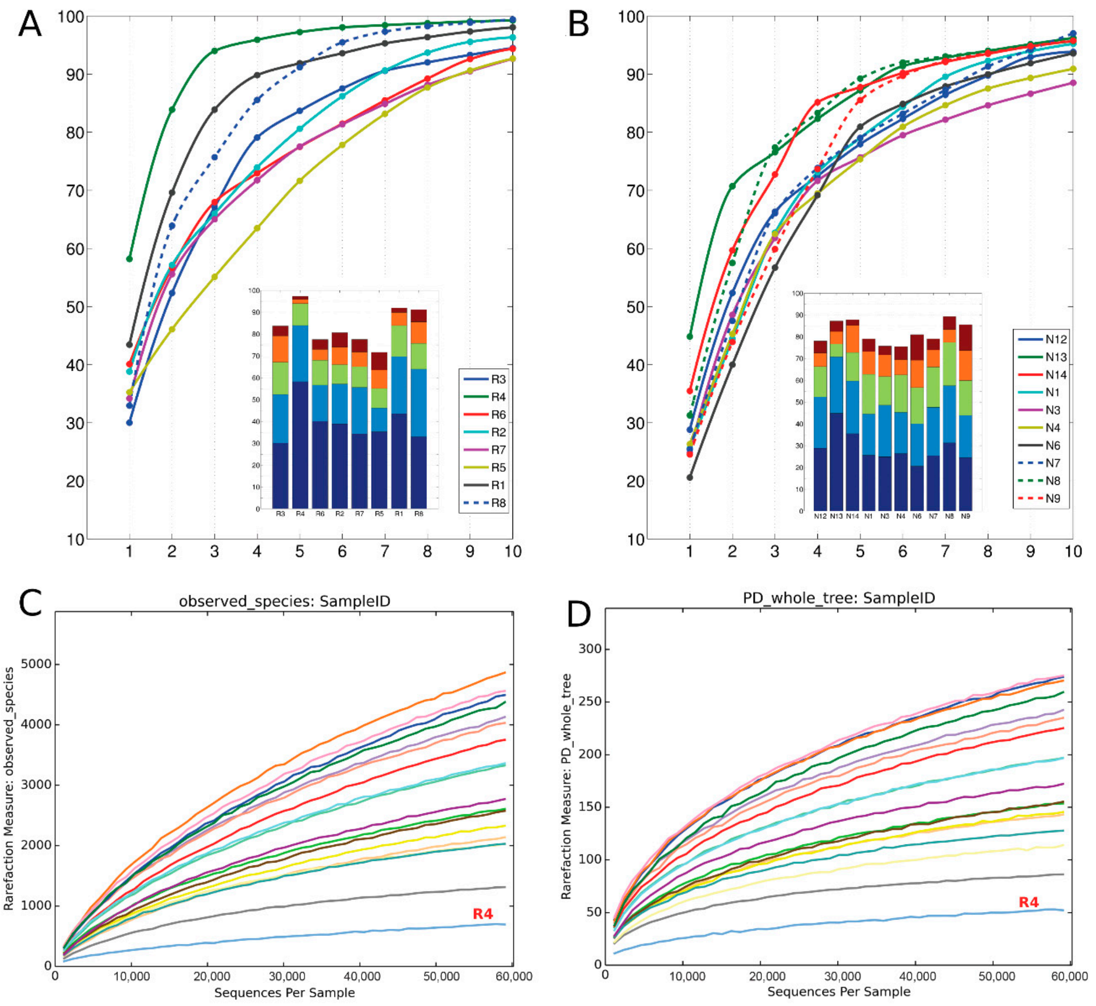

Figure S2. Relative abundances for most abundant families represented as a cumulative sum. For all the Rett (RTT, **A**) and controls (CTR, **B**) samples, the most abundant 10 family groups were summed up sequentially. In each panel, the inset shows the stacked column chart of the main 5 families. Observed species (**C**) and Faith's phylogenetic tree distances (**D**) rarefaction curves of α diversity measures for all samples (various colors), highlighting the very low biodiversity of the outlier R4 sample.

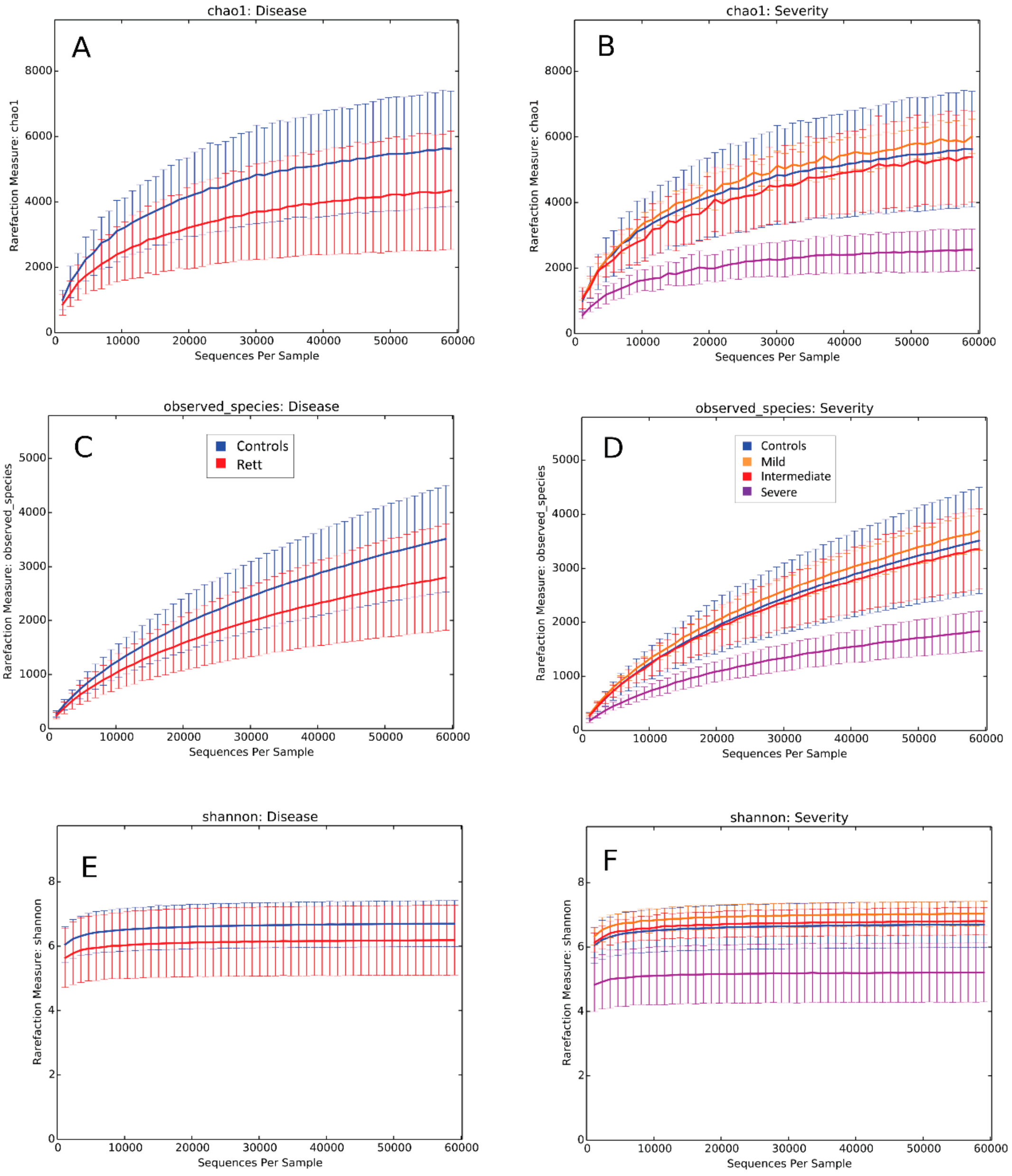

Figure S3. α-diversity rarefaction curves for non-phylogenetic indexes: chao1 (**A**,**B**), observed species (**C**,**D**) and Shannon diversity index (**E**,**F**). (**A**,**C**,**E**) group samples according to disease status (Rett–Control), whereas (**B**,**D**,**F**) group samples according to disease severity. All differences between groups are not statistically significative ($p > 0.05$, permutation-based non-parametric *t*-test).

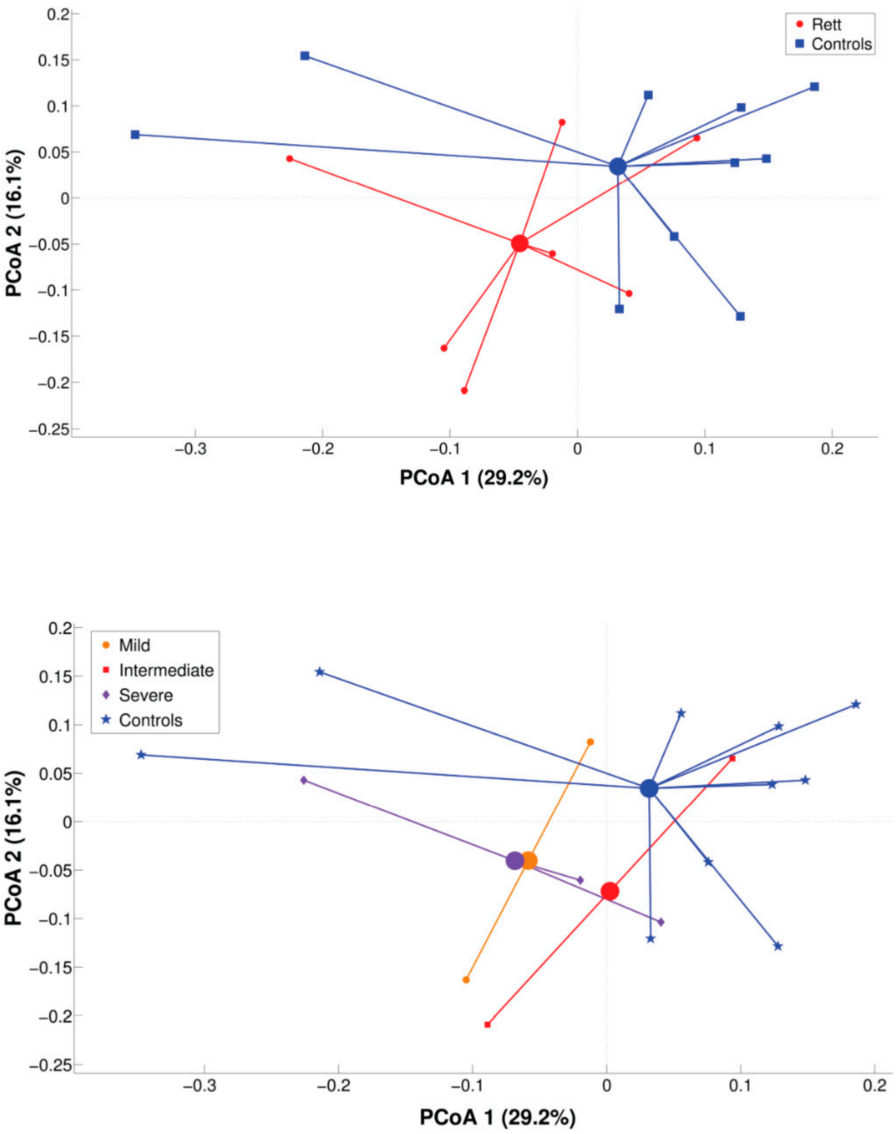

Figure S4. Principal coordinate analysis (PCoA) according to Weighted Unifrac distance. The first two components of the variance are represented. (**A**) PCoA of Rett (red) versus control (blue) samples. The two groups separate according PCoA 1 component. Differences are not statistically significant ($p > 0.05$); (**B**) Samples are colored according to disease's severity. Severe stage (purple) significantly ($p < 0.05$) separates from healthy controls. Other differences are not statistically significant ($p > 0.05$).

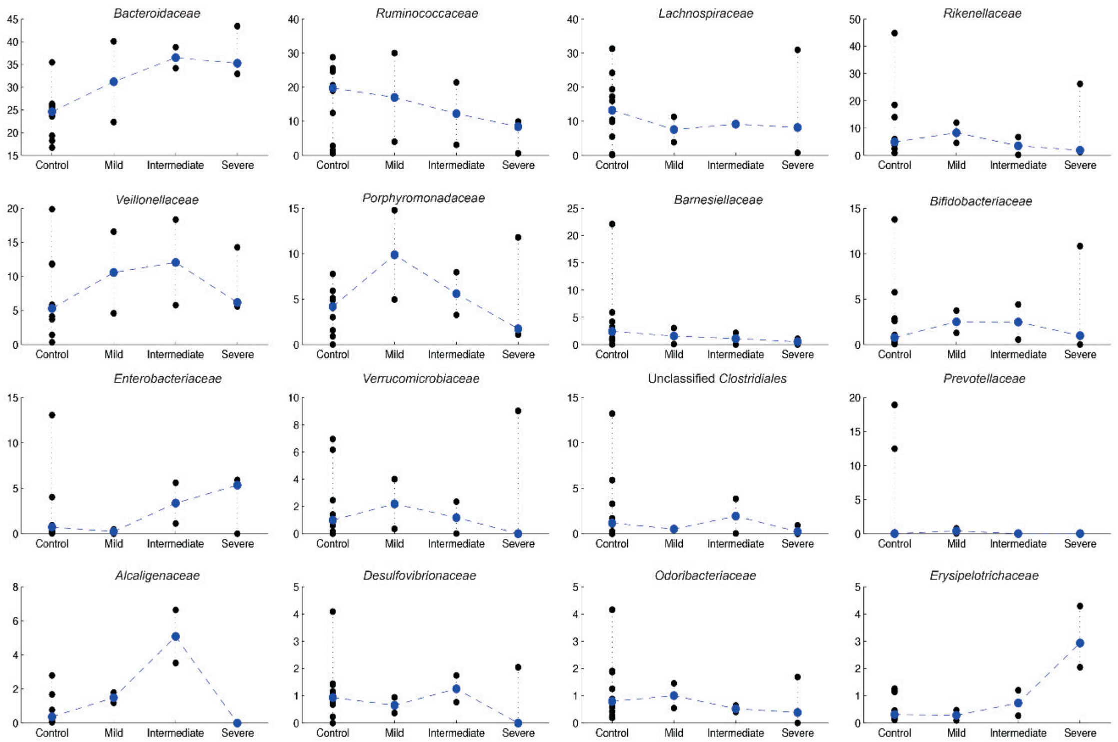

Figure S5. Trends of family relative abundances categorized according the severity of Rett phenotype.

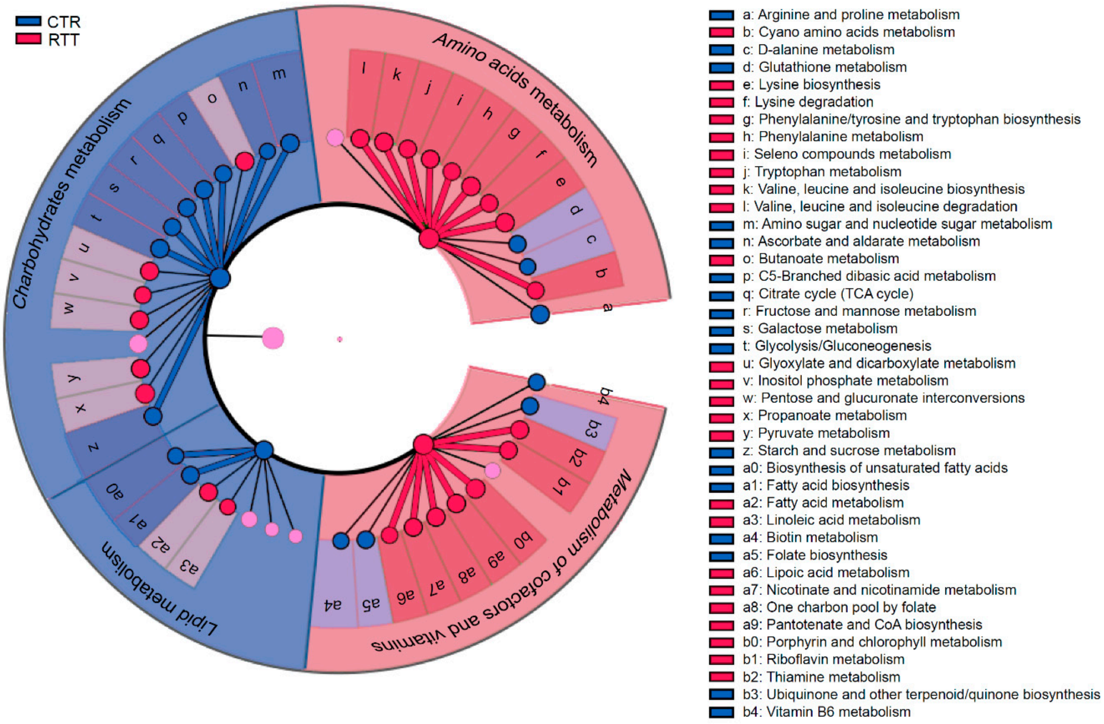

Figure S6. Kyoto Encyclopedia of Genes and Genomes (KEGG) pathways differentially abundant in Rett (RTT, magenta) and control (CTR, blue) gut microbiota. Cladogram represents the KEGG BRITE functional hierarchy: the outermost circles represent very broad functional categories, and innermost specific metabolic pathways.

Table S1. Diet-microbiota correlation.

Taxonomic Level	Taxon	Avg CTR	Avg RTT	kcal/Day	Protein	Animal Proteins	Vegetal Proteins	Chloresterol	Fibers	Fats
Phylum	*Proteobacteria*	4.6	5.8	-	-	0.52434	-	-	-	-
	Tenericutes	0.2	0	-	-	-	-	-	0.62203	-
Family	*Bacteroidaceae*	24.1	35.3 *	0.55375	0.61466	0.53638	0.49613	-	-	-
	Porphyromonadaceae	3.8	6.5	-	-	-	-	-0.51745	-	-
	Alcaligenaceae	0.7	1.9	0.4964	0.65662	0.72738	0.58491	-	-	-
	Erysipelotrichaceae	0.5	1.6	0.51127	-	-	-	-0.62707	-	-
	Unclassified *Clostridiales*	2.7	0.9	-	-	-	-	-	0.64426	-
	Odoribacteraceae	1.2	0.7	-	-	-	-	-	-	0.50782
	Pseudomonadaceae	0	0.3	0.55261	0.66264	0.71857	0.57819	-	-	-
	Christensenellaceae	0.5	0.1	-	-	-	-	-	0.50341	-
Genus	*Bacteroides*	24.1	35.3 *	0.55374	0.61465	0.53636	0.49612	-	-	-
	Ruminococcus	3.8	2.9	0.62187	-	-	-	-0.51042	-	-
	Acidaminococcus	1.3	2.8	-	-	0.53791	-	-	-	-
	Ruminococcus	0.3	2.7	0.61614	-	-	-	-0.58799	-	-
	Clostridium	0.4	2.2 **	-	0.63432	0.60622	-	-0.62134	-	-
	Megasphaera	0	2.1	0.50076	0.62374	0.68059	0.59154	-	-	-
	Sutterella	0.7	1.9	0.4966	0.65659	0.72737	0.58469	-	-	-
	Escherichia	0.7	1.7	-	0.54401	0.60502	-	-	-	-
	Unclassified *Clostridiales*	2.7	0.9	-	-	-	-	-	0.64426	-

Pearson correlation coefficients between macronutrients (expressed as normalized quantities on the individual body weight) and relative abundances of microbiota profiles at phylum, family and genus level. Only significant ($p < 0.05$) correlations are reported. Average relative abundances on RTT and CTR datasets for the main bacterial groups are also reported, as well as the significativity of the Mann–Whitney test on relative abundance. Significant differences are indicated by * $p < 0.05$, and ** $p < 0.01$.
